# Lifestyle Behaviors and Self-Rated Health: The Living for Health Program

**DOI:** 10.1155/2014/315042

**Published:** 2014-10-28

**Authors:** Gustavo G. Zarini, Joan A. Vaccaro, Maria A. Canossa Terris, Joel C. Exebio, Laura Tokayer, Janet Antwi, Sahar Ajabshir, Amanpreet Cheema, Fatma G. Huffman

**Affiliations:** ^1^Department of Dietetics and Nutrition, Robert Stempel College of Public Health and Social Work, Florida International University, 11200 SW 8th Street, AHC-5 307, Miami, FL 33199, USA; ^2^Florida Heart Research Institute, 4770 Biscayne Boulevard 5th Floor, Miami, FL 33137, USA; ^3^Department of Dietetics and Nutrition, Robert Stempel College of Public Health and Social Work, Florida International University, 11200 SW 8th Street, AHC-5 306, Miami, FL 33199, USA

## Abstract

*Background*. Lack of adherence to dietary and physical activity guidelines has been linked to an increase in chronic diseases in the United States (US). The aim of this study was to assess the association of lifestyle behaviors with self-rated health (SRH). *Methods*. This cross-sectional study used self-reported data from Living for Health Program (*N* = 1,701) which was conducted from 2008 to 2012 in 190 health fair events in South Florida, US. *Results*. Significantly higher percent of females as compared to males were classified as obese (35.4% versus 27.0%), reported poor/fair SRH (23.4% versus 15.0%), and were less physically active (33.9% versus 25.4%). Adjusted logistic regression models indicated that both females and males were more likely to report poor/fair SRH if they consumed ≤2 servings of fruits and vegetables per day (OR = 2.14, 95% CI 1.30–3.54; OR = 2.86, 95% CI 1.12–7.35, resp.) and consumed mostly high fat foods (OR = 1.58, 95% CI 1.03–2.43; OR = 3.37, 95% CI 1.67–2.43, resp.). The association of SRH with less physical activity was only significant in females (OR = 1.66, 95% CI 1.17–2.35). *Conclusion*. Gender differences in health behaviors should be considered in designing and monitoring lifestyle interventions to prevent cardiovascular diseases.

## 1. Introduction

Self-evaluation of general health status has been associated with actual health in that what people report about their health has been shown to predict mortality [1]. Self-rated health (SRH) as a single survey question developed by the World Health Organization (WHO) [2] has been validated as a tool to predict mortality in populations with and without cardiovascular diseases [1–3] and functional ability [4, 5]. Asking participants to describe their overall health on a five-point scale (ranging from excellent to poor) has achieved popularity as a health-indicating tool in the United States (US) and other countries [6, 7].

Health is largely influenced by modifiable risk factors such as diet and physical activity [8]. Noncommunicable diseases are the leading causes of death globally [9]. Worldwide, noncommunicable diseases (cancer, cardiovascular diseases, diabetes, and chronic lung diseases) are largely attributed to four main behavioral factors: tobacco use, unhealthy diet, insufficient physical activity, and harmful alcohol use [9]. Poor health for persons with chronic diseases has been attributed largely to a lack of adherence to medical recommendations which include diet and physical activity. High consumption of fruits and vegetables resulted in a lower incidence of cardiovascular disease in nurses and health professionals in a 14-year follow-up study [10]. Reporting poor health may be due, in part, to poor management of chronic disease. Symptoms of chronic diseases attributed to poor SRH among a Swedish population of middle-aged and older adults were tiredness/weakness, depression, and musculoskeletal pains [11].

Lifestyle behaviors, particularly diet and physical activity, can contribute to or help prevent cardiovascular diseases such as coronary heart disease, hypertension, and type 2 diabetes [8]. Diet and physical activity can have positive effects for persons with preexisting coronary heart disease, as well [12]. Reduction in chronic disease burden and increase in quality of life have been well documented with higher daily consumption of fruits and vegetables [13, 14] and physical activity [15]. The relative risk of cardiovascular disease events for persons with type 2 diabetes decreased with moderate to vigorous physical activity in 14-year follow-up studies for men [16] and women [17] even after adjusting for sociodemographic factors. Moreover, SRH, as an indicator of health and wellbeing, has been positively associated with fruits and vegetables intake [18, 19]. Eating at fast-food restaurants [20] and high-fat diets [21] were associated with poor/fair SRH in African-Americans and Australian women, respectively. Following dietary and physical activity recommendations has been associated with the prevention and lessening of the severity of cardiovascular diseases [8]. Dietary recommendations include half your plate being comprised of fruits and vegetables and cutting back on foods high in solid fats [22]. Physical activity guidelines specify being physically active most days of the week to attain 150 minutes of moderate or 75 minutes of vigorous physical activity per week [22].

Lack of physical activity and unhealthy dietary patterns place a burden on society due to lack of work productivity and increased health care costs [23]. Physical health components from the Short Form 12 (SF-12) were associated with increased physical activity and healthier diet from baseline to five-year follow-up of a European cohort from the Inter99 study [24]. Several studies have found an association with lifestyle behaviors and self-evaluation of health. In particular, regular physical activity is reported to be significantly associated with SRH in various populations [23–26]. Adults from the US with and without cardiovascular diseases who reported engaging in healthy dietary and physical activity behaviors had a higher odds of reporting optimal SRH (excellent, very good, or good) as compared to fair or poor [27]. Physical activity, besides lowering the risk for the development of chronic diseases, also improves the health outcomes of such disease conditions [28]. Therefore, the objective of this study was to examine the relationship between dietary and physical activity behaviors and SRH. It was hypothesized that participants who reported lower fruits and vegetables intake and higher fatty-food intake and perform less physical activity will have SRH poor/fair as compared to those in the good, very good, or excellent category, adjusting for covariates.

## 2. Materials and Methods

### 2.1. Sample

This is a secondary data analysis of the Living for Health Program (Florida Heart Research Institute, Miami, Florida, US). The Living for Health Program was conducted from 2008 to 2012 and collected information on health behaviors from adults ≥18 years old (*N* = 9,453). During these years, 190 health fair events were held in low income minority communities in Miami-Dade County to screen participants for cardiovascular disease risks. An accredited Institutional Review Board (IRB) provided a waiver of consent for the analyses. Self-rated health assessment was added in the year 2012 and was answered by 2,108 individuals during the remainder of the Living for Health Program. The final sample size (*N* = 1,701) consisted of the participants who responded to all the questions included in this analysis.

### 2.2. Measures

#### 2.2.1. Self-Rated Health and Sociodemographic Information

Self-rated health was measured by the following question: “how would you rate your current health state?” There were five possible responses: excellent, very good, good, fair, and poor. The responses were categorized into two as follows: (1) excellent/very good/good or (2) fair/poor for this study. The sociodemographic questionnaire collected information on age, gender, ethnicity, smoking, health insurance, and comorbidities. Age was converted to a categorical variable (18–30, 31–55, and >55 years old) to assess differences between older and younger adults based on the demographics of the population. Smoking status derived from the question “do you smoke (any tobacco product)?” and was categorized as (yes/no). There were insufficient participants to access differences for the original five possible categories for ethnicity. Based on the distribution, ethnicity was collapsed into three categories: Hispanic, non-Hispanic Black, and other. Other category included White, American Indian, Pacific Islander, Indian, and Asian. Health insurance was categorized as a binary variable (no insurance/yes insurance). Presence of comorbidities (yes/no) was measured combining two questions: (1) “has any medical professional ever told you that you have high glucose, high blood pressure, high cholesterol, coronary heart disease)” and (2) “have you been prescribed medications for (diabetes, hypertension, cholesterol, coronary heart disease)?” A positive response to either question was categorized as having comorbidities.

#### 2.2.2. Anthropometrics Measures

Participant's height and weight were measured and body mass index (BMI) was calculated as weight (kg)/height (m^2^). BMI was classified as BMI: 18.5–29.9 m^2^ (nonobese) and BMI: ≥30 (obese). There were <1.0% of participants classified as underweight (BMI <18.5); these participants were included in as part of the “nonobese” group.

#### 2.2.3. Fruits and Vegetables Intake

A single question was used to measure fruits and vegetables intake: “on a typical day in the past month, how many servings of fruits and vegetables did you eat?” Examples of what a serving represents were provided with the question (i.e., 1 cup fresh, 1 medium size fruit, etc.). The five possible options were 5 or more per day, 4 a day, 3 a day, 2 a day, or 1 a day. The responses were categorized into three as follows: (1) 5 or more per day; (2) 3-4 per day; (3) 2 or less per day.

#### 2.2.4. Fat Intake

Fat intake was measured by the question: “if you ate fast food in the last month, what type was it?” Examples of high fat foods (fried food, breaded items, taco salads, nachos, double burgers, pizza, hot dogs, croissant items, donuts, shakes, and cakes) and low fat foods (salads (no creamy dressings), single burgers, grilled chicken, fruits, and yogurt parfaits) were provided. From the five possible options, the responses were categorized into three as follows: (1) primarily low fat foods, mostly low fat or some high fat; (2) both high fat and low fat foods about the same; (3) mostly high fat, some low fat or primarily high fat foods.

#### 2.2.5. Physical Activity

Physical activity was measured with the question: “in the last month, how often were you physically active?” (performance of at least 30 minutes of fitness walking, cycling, jogging, swimming, aerobic dance, or active sports was considered physical activity). Physical activity was collapsed from six possible options into three categories as follows: (1) 5–7 days a week; (2) 1–4 days a week; (3) 1–3 times a month or never to allow sufficient number in each category.

#### 2.2.6. Statistical Analysis

The general characteristics of the participants were examined with descriptive statistics. Differences between male and female participants were assessed with the Chi-square test for categorical variables. Logistic regression models were conducted to examine the relationship between the binary dependent variable, SRH with fruits and vegetables intake, physical activity, and fat intake adjusting for confounding variables. Covariates were considered potential confounders based on the literature. Age and gender differences were found in perceptions of SRH [11, 29]. Poorer physical SRH was also associated with lack of health insurance, minority status, and less education for a cohort of randomly selected Kentucky adults [30]. Comorbidities and obesity are known factors of poorer health. Since gender interacted with SRH and with physical activity, models were performed by splitting data by gender. For each lifestyle behavior (fruit and vegetable intake, fat intake, and physical activity) a reduced model (unadjusted) and full model (adjusted) including covariates (age, BMI, smoking status, ethnicity, health insurance, and comorbidities) were conducted. The significance level for all analyses was set at *P* < 0.05. All statistical analyses were performed using IBM Statistical Package for the Social Science version 19 (SPSS Inc., Chicago IL, US).

## 3. Results

Table 1 compared general characteristics between males and females. Females as compared to males had a higher percent classified as obese (35.4% versus 27.0%, *P* = 0.001), reported poor/fair SRH (23.4% versus 15.0%, *P* < 0.001), and were less physically active (33.9% versus 25.4%, *P* = 0.001). Participants who reported poor/fair SRH as compared to those with excellent/very good/good were females (78.6% versus 67.9%, *P* < 0.001); older > 55 years (35.5% versus 29.4%, *P* = 0.024); obese (46.8% versus 29.2%, *P* < 0.001); non-Hispanic Black (38.0% versus 29.4%, *P* = 0.001); had comorbidities (61.4% versus 40.5%, *P* < 0.001); consumed ≤2 servings of fruits and vegetables per day (73.0% versus 59.3%, *P* < 0.001); had an intake of primary high fat foods/mostly high fat or some low fat foods (17.7% versus 10.8%, *P* < 0.001); and were less physically active (40.3% versus 29.0%, *P* < 0.001) (Table 2).

### 3.1. Fruits and Vegetables Intake and Self-Rated Health

Unadjusted logistic regression models for females showed that those who consumed ≤2 servings of fruits and vegetables per day were 2.2 times more likely to report poor/fair SRH as compared to those who consumed ≥5 servings per day (*P* = 0.001, 95% CI 1.39–3.70). Logistic regression model adjusted for age, BMI, smoking, ethnicity, health insurance, and comorbidities showed that females who consumed ≤2 servings of fruits and vegetables per day were 2.1 times more likely to report poor/fair SRH as compared to those who consumed ≥5 servings per day (*P* = 0.003, 95% CI 1.23–3.54). The adjusted model explained 11.8% of the variation in SRH (Table 3).

There was a more modest and nonstatistically significant increase in the odds of poor/fair SRH for men with the lowest level of daily fruits and vegetables consumption. Unadjusted and adjusted models indicated that males who consumed 3-4 servings of fruits and vegetables per day were 2.6 and 2.8 times more likely to report poor/fair SRH as compared to those who consumed ≥5 servings per day (*P* = 0.031, 95% CI 1.09–6.58 and *P* = 0.028, 95% CI 1.12–7.35, resp.). The model explained 12.2% of the variation in SRH (Table 4).

### 3.2. Fat Intake and Self-Rated Health

Unadjusted logistic regression models of fat intake for females indicated that those who consumed mostly high fat foods were 1.6 times more likely to report poor/fair SRH as compared to those who consumed primarily low fat foods (*P* = 0.015, 95% CI 1.103–2.49). Females who consumed both high and low fat foods were 1.3 times more likely to report poor/fair SRH as compared to those who consumed primarily low fat foods (*P* = 0.041, 95% CI 1.013–1.83). Logistic regression models adjusted for age, BMI, smoking, ethnicity, health insurance, and comorbidities showed that females who consumed mostly high fat foods were 1.5 times more likely to report poor/fair SRH as compared to those who had primarily low fat foods (*P* = 0.036, 95% CI 1.03–2.43). Similarly, females who consumed both high and low fat foods about the same were 1.4 times more likely to report poor/fair SRH as compared to those who had primarily low fat foods (*P* = 0.018, 95% CI 1.07–2.01). The model explained 10.0% of the variation in SRH (Table 3).

Unadjusted logistic regression model for males indicated that those who consumed mostly high fat foods were 3.1 times more likely to report poor/fair SRH as compared to those who consumed primarily low fat foods (*P* < 0.001, 95% CI 1.70–5.97). The adjusted model showed that males who consumed mostly high fat foods were 3.2 times more likely to report poor/fair SRH health as compared to those who consumed primarily low fat foods (*P* = 0.001, 95% CI 1.67–2.43). The model explained 14.1% of the variation in SRH (Table 4).

### 3.3. Physical Activity and Self-Rated Health

Unadjusted logistic regression model for females showed that those who performed 1–3 times a month or never of physical activity were 1.6 times more likely to report poor/fair SRH as compared to those who performed ≥5 days per week of physical activity (*P* = 0.003, 95% CI 1.18–2.29). Logistic regression model adjusted for age, BMI, smoking, ethnicity, health insurance, and comorbidities showed that females who reported 1–3 times a month or never of physical activity were 1.6 times more likely to report poor/fair SRH as compared to females who performed ≥5 days per week of physical activity (*P* = 0.004, 95% CI 1.17–2.35). The model explained 10.3% of the variation in SRH (Table 3).

Unadjusted logistic regression model showed that males who performed 1–3 times a month or never of physical activity were 1.8 times more likely to report poor/fair SRH as compared to those who performed ≥5 days per week of physical activity (*P* = 0.043, 95% CI 1.02–3.40). The association of physical activity and SRH was no longer significant for males once adjustment variables were added (*P* = 0.218, OR = 1.50, 95% CI 0.79–2.49) (Table 4).

## 4. Discussion

### 4.1. Fruits and Vegetables Intake and Self-Rated Health

We found higher odds of poor/fair SRH for males and females who consumed the lowest level of fruits and vegetables. No gender differences were found in a longitudinal cohort of Canadian young adults who were more likely to report excellent SRH if they had a high intake of fruits and vegetables as adolescents [31]. Similarly, dietary intake high in fiber was associated with more favorable SRH as compared to lower fiber for a cohort of African-American adults, independent of gender [32]. There are several potential confounders to the association of diet and health. The presence of any chronic disease influences one's perception of health and wellbeing [33–35]. Persons with diabetes may be more likely to consume more fruits and vegetables after their diagnosis [27]. Even though diagnosis with chronic disease could result in better health behavior, consuming fruits and vegetables at least five times per day was associated with optimal SRH for both individuals with cardiovascular diseases and diabetes as well as for persons without these chronic illnesses [27]. Our sample population, who are approximately more than half-Hispanic, may have a large proportion of first generation immigrants from Caribbean and Latin American countries. The perception of good health may differ based on country of origin. For example, foreign-born Haitian Americans had higher levels of perceived stress as compared to African-Americans, but African-Americans were more likely to rate their health as poor/fair compared to Haitian Americans [36].

### 4.2. Fat Intake and Self-Rated Health

We found that both males and females, who consumed mostly high fat foods, were more likely to report poor/fair SRH as compared to those consuming primarily low fat foods. High fat intake was also associated with poorer SRH in African-American adults [32]. Consistent with our results, Collins et al. [21] reported higher intake of fat and saturated fat to be associated with poorer SRH for a large cohort of Australian women. Frequency of eating at fast-food restaurants was positively associated with poor SRH for a cohort of African-Americans [20]. Our results contradicted Barreto and de Figueiredo [33] who reported lower odds of consuming fatty meat and whole milk for those diagnosed with at least one chronic disease and even lower for those diagnosed with two or more chronic diseases compared to absence of disease in a Spanish population. They suggested that medical advice to modify lifestyle behavior may be a probable confounder of diet and health when comparing persons with and without chronic disease [33]. Similarly, the odds of having poor health increased twofold in a cohort of Greek nurses making an effort to avoid fatty foods in their diet [37].

### 4.3. Physical Activity and Self-Rated Health

A strong association between physical activity and SRH for females was observed in our study. Females who engaged in less physical activity were more likely to report poor/fair SRH as compared to those who were more physically active. Several studies found physical activity to be related to SRH regardless of gender for a national sample [27], for a cohort of older adults [26], and for African-American adults with a high proportion of chronic diseases [32]. Our findings on gender differences in physical activity levels and SRH are in agreement with several other studies [38–41]. The propensity to be less physically active was prevalent among women in our sample. As expected, the likelihood of rating health as poor/fair was substantially higher for women, as compared to men. Considering the impact of physical activity on health status, variation of the effect of physical activity according to gender could also be associated with different proportions of SRH. These distinctions could be explained by the biological and sociocultural environment inequalities between men and women. For instance, it was found that gender roles and reduced access to resources and social conditions, such as safe environment, do not foster regular physical activity among women [40, 41]. While women generally tend to rate their health worse in health and behavioral studies, this is consistently in line with lack of physical activity. Of utmost relevance of the role of physical activity in health is the gradient effect of the levels of physical activity on SRH, as demonstrated by evidence from several past studies [42–44]. Participants who were highly active and very highly active were twice as likely to have excellent SRH as compared to those who were less physically active [43].

### 4.4. Self-Rated Health and Ethnicity

The main objective of our study was to assess the relationship of indicators of modifiable cardiovascular disease risk factors as follows: dietary and physical activity lifestyle and their relationship to SRH. We examined SRH in a half-Hispanic population with non-Hispanics who were predominately Black and we found significant differences in ethnicity for SRH. However, it is noteworthy to mention that ethnic differences were adjusted in the final models. Several studies indicated that ethnicity was a factor in the assessment of SRH. A cohort of older Hispanics rated their health poorer than non-Hispanic Whites yet had a lower mortality rate after a 16-year follow-up, based on data from the Health and Retirement Study and adjusting for demographics, socioeconomic status, health status, and health behaviors at baseline [45]. Furthermore, level of acculturation in Hispanic Americans was associated with SRH whereby SRH of the more acculturated matched native non-Hispanic Whites and Blacks [46]. Self-rated health differences were found within African-Americans over time that were based on differences in age, education, smoking, and morbidity (angina, congestive heart failure, diabetes, and kidney disease), having been hospitalized in the year prior to baseline, depressive symptoms, mobility limitations, and initial self-rated health [47].

### 4.5. Strengths and Limitations

There were several strengths of this study. This was a large sample of adults from Miami Dade County, Florida, primarily minorities, half of whom were from a diverse Hispanic population. To our knowledge, this is the first study to investigate the relationship between lifestyle behaviors and SRH for a population largely comprised of Hispanics with a considerable proportion of Blacks. Nevertheless, there are some limitations. Causality of lifestyle behaviors with SRH could not be established due to the single time point. The measurements were self-reported and may be over- or underestimated. Individuals reporting good SRH may have overstated healthy eating and physical activity level. Other factors that may have influenced SRH such as socioeconomic status, neighborhood, housing situation, and psychosocial factors were not collected in this study.

## 5. Conclusion

Modifiable lifestyle behaviors known to reduce cardiovascular disease risk, low fruits and vegetables intake and high fat intake, were associated with poor/fair SRH in males and females. Poorer SRH was significantly associated with low physical activity in females only, and low physical activity was also more common in women than men in this study sample. These results indicate that gender differences may have implications in designing and monitoring lifestyle interventions to prevent cardiovascular diseases.

## Figures and Tables

**Table 1:** Characteristics of the study participants, total and by gender.

Variables	Total sample (*n* = 1,701)	Male (*n* = 508)	Female (*n* = 1,193)	*P* value^†^
Age (years)				
18–30	286 (16.8)	98 (19.3)	188 (15.8)	0.075
31–55	893 (52.5)	247 (48.6)	646 (54.1)	
>55	522 (30.7)	163 (32.1)	359 (30.1)	
Body mass index (BMI)				0.001
<29.9 kg/m^2^	1,142 (67.1)	371 (73.0)	771 (64.6)	
≥30 kg/m^2^	559 (32.9)	137 (27.0)	422 (35.4)	
Smoke				<0.001
No	1,559 (91.7)	445 (87.6)	1,114 (93.4)	
Yes	142 (8.3)	63 (12.4)	79 (6.6)	
Ethnicity				0.073
Hispanic	942 (55.4)	291 (57.3)	651 (54.6)	
Non-Hispanic Black	531 (31.2)	140 (27.6)	391 (32.8)	
Other	228 (13.4)	77 (15.2)	151 (12.7)	
Health insurance				0.454
No	1,041 (61.2)	304 (59.8)	737 (61.8)	
Yes	660 (38.8)	204 (40.2)	456 (38.2)	
Comorbidities				0.464
No	938 (55.1)	287 (56.5)	651 (54.6)	
Yes	763 (44.9)	221 (43.5)	542 (45.4)	
Self-rated health				
Poor/fair	355 (20.9)	76 (15.0)	279 (23.4)	<0.001
Excellent/very good/good	1,346 (79.1)	432 (85.0)	914 (76.6)	
F & V (servings/day)				
≤2 per day	1,057 (62.1)	332 (65.4)	725 (60.8)	0.187
3-4 per day	453 (26.6)	126 (24.8)	327 (27.4)	
≥5 per day	191 (11.2)	50 (9.8)	141 (11.8)	
Fat intake				0.377
Primary high fat foods/mostly high fat, some low fat	209 (12.3)	69 (13.6)	140 (11.7)	
Both high fat and low fat about the same	538 (31.6)	166 (32.7)	372 (31.2)	
Mostly low fat, some high fat/primary low fat foods	954 (56.1)	273 (53.7)	681 (57.1)	
Physical activity				0.001
1–3 times per month or never	533 (31.3)	129 (25.4)	404 (33.9)	
1–4 days per week	599 (35.2)	186 (36.6)	413 (34.6)	
≥5 days per week	569 (33.5)	193 (38.0)	376 (31.5)	

F & V = fruits and vegetables. ^†^Statistical differences by gender: Chi-square test. Data are presented as *n* (%). *P* value is considered significant at <0.05.

**Table 2:** Characteristics of the study participants by self-rated health.

Variables	Self-rated health		*P* value
	Poor/fair (*n* = 355)	Excellent/very good/good (*n* = 1,346)	
Gender			<0.001
Men	76 (21.4)	432 (32.1)	
Women	279 (78.6)	914 (67.9)	
Age (years)			
18–30	46 (13.0)	240 (17.8)	0.024
31–55	183 (51.0)	710 (52.7)	
>55	126 (35.5)	396 (29.4)	
Body mass index (BMI)			<0.001
<29.9 kg/m^2^	189 (53.2)	953 (70.8)	
≥30 kg/m^2^	166 (46.8)	393 (29.2)	
Smoke			0.610
No	323 (91.0)	1236 (91.8)	
Yes	32 (9.0)	110 (8.2)	
Ethnicity			0.001
Hispanic	165 (46.5)	777 (57.7)	
Non-Hispanic Black	135 (38.0)	396 (29.4)	
Other	55 (15.5)	173 (12.9)	
Health insurance			0.054
No	233 (65.6)	808 (60.0)	
Yes	122 (34.4)	538 (40.0)	
Comorbidities			<0.001
No	137 (38.6)	801 (59.5)	
Yes	218 (61.4)	545 (40.5)	
F & V (servings/day)			<0.001
≤2 per day	259 (73.0)	798 (59.3)	
3-4 per day	64 (18.0)	389 (28.9)	
≥5 per day	32 (9.0)	159 (11.8)	
Fat intake			<0.001
Primary high fat foods/mostly high fat, some low fat	63 (17.7)	146 (10.8)	
Both high fat and low fat about the same	119 (33.5)	419 (31.1)	
Mostly low fat, some high fat/primary low fat foods	173 (48.7)	781 (58.0)	
Physical activity			<0.001
1–3 times per month or never	143 (40.3)	390 (29.0)	
1–4 days per week	114 (32.1)	485 (36.0)	
≥5 days per week	98 (27.6)	471 (35.0)	

F & V = fruits and vegetables. Statistical differences by self-rated health: Chi-square test. Data are presented as *n* (%). *P* value is considered significant at <0.05.

**Table 3:** Logistic regression: unadjusted and adjusted associations between lifestyle behaviors and poor/fair self-rated health for females.

Variables	Unadjusted			Adjusted^†^		
	OR	95% CI for OR	*P* value	OR	95% CI for OR	*P* value
F & V (servings/day)						
≥5 per day	**Reference**	—	—	**Reference**	—	—
3-4 per day	1.08	0.62–1.87	0.783	1.03	0.59–1.82	0.907
≤2 per day	2.27	1.39–3.70	0.001	2.14	1.30–3.54	0.003
Fat intake						
Mostly low fat, some high fat/primary low fat foods	**Reference**	—	—	**Reference**	—	—
Both high fat and low fat about the same	1.36	1.01–1.83	0.041	1.46	1.07–2.01	0.018
Primary high fat foods/mostly high fat, some low fat	1.66	1.10–2.49	0.015	1.58	1.03–2.43	0.036
Physical activity						
≥5 days per week	**Reference**	—	—	**Reference**	—	—
1–4 days per week	1.12	0.79–1.58	0.517	1.08	0.75–1.55	0.663
1–3 times per month or never	1.64	1.18–2.29	0.003	1.66	1.17–2.35	0.004

F & V = fruits and vegetables; OR = odds ratio; CI = confidential interval. ^†^The other covariates included in the models were age, BMI, smoking, ethnicity, health insurance, and comorbidities.

**Table 4:** Logistic regression: unadjusted and adjusted associations between lifestyle behaviors and poor/fair self-rated health for males.

Variables	Unadjusted			Adjusted^†^		
	OR	95% CI for OR	*P* value	OR	95% CI for OR	*P* value
F & V (servings/day)						
≥5 per day	**Reference**	—	—	**Reference**	—	—
3-4 per day	2.68	1.09–6.58	0.031	2.86	1.19–7.35	0.028
≤2 per day	1.48	0.71–3.09	0.289	1.57	0.72–3.42	0.257
Fat intake						
Mostly low fat, some high fat/primary low fat foods	**Reference**	—	—	**Reference**	—	—
Both high fat and low fat about the same	1.11	0.62–1.98	0.721	1.202	0.65–2.21	0.554
Primary high fat foods/mostly high fat, some low fat	3.18	1.70–5.97	<0.001	3.367	1.69–2.43	0.001
Physical activity						
≥5 days per week	**Reference**	—	—	**Reference**	—	—
1–4 days per week	1.09	0.60–1.99	0.771	0.83	0.44–1.56	0.562
1–3 times per month or never	1.86	1.02–3.40	0.043	1.50	0.79–2.49	0.218

F & V = fruits and vegetables; OR = odds ratio; CI = confidential interval. ^†^The other covariates included in the models were age, BMI, smoking, ethnicity, health insurance, and comorbidities.
